# Impact of Surface-Active Guanidinium-, Tetramethylguanidinium-, and Cholinium-Based Ionic Liquids on *Vibrio Fischeri* Cells and Dipalmitoylphosphatidylcholine Liposomes

**DOI:** 10.1038/srep46673

**Published:** 2017-04-21

**Authors:** Antti H. Rantamäki, Suvi-Katriina Ruokonen, Evangelos Sklavounos, Lasse Kyllönen, Alistair W. T. King, Susanne K. Wiedmer

**Affiliations:** 1 Department of Chemistry, POB 55, 00014 University of Helsinki, Finland; 2 Kemira Oyj, Luoteisrinne 2, POB 44, 02271 Espoo, Finland

## Abstract

We investigated the toxicological effect of seven novel cholinium, guanidinium, and tetramethylguanidinium carboxylate ionic liquids (ILs) from an ecotoxicological point of view. The emphasis was on the potential structure-toxicity dependency of these surface-active ILs in aqueous environment. The median effective concentrations (EC_50_) were defined for each IL using *Vibrio (Aliivibrio) fischeri* marine bacteria. Dipalmitoylphosphatidylcholine (DPPC) liposomes were used as biomimetic lipid membranes to study the interactions between the surface-active ILs and the liposomes. The interactions were investigated by following the change in the DPPC phase transition behaviour using differential scanning calorimetry (DSC). Critical micelle concentrations for the ILs were determined to clarify the analysis of the toxicity and the interaction results. Increasing anion alkyl chain length increased the toxicity, whereas branching of the chain decreased the toxicity of the ILs. The toxicity of the ILs in this study was mainly determined by the surface-active anions, while cations induced a minor impact on the toxicity. In the DSC experiments the same trend was observed for all the studied anions, whereas the cations seemed to induce more variable impact on the phase transition behaviour. Toxicity measurements combined with liposome interaction studies can provide a valuable tool for assessing the mechanism of toxicity.

Ionic liquids (ILs) are organic salts that are typically in the molten state at temperatures below 100 °C. ILs have been considered as “green” alternatives for molecular solvents, since there is lower risk of environmental release through the atmosphere due to their low vapour pressures. However, because of their water solubility, ILs may be a potent environmental hazard and therefore a danger to living organisms from microbes to humans. The risks remain throughout the whole life cycle of the ILs, from synthesis to application and from application to disposal. Potential hazards have been reviewed for instance by Cvjetko Bubalo *et al*.^1^, Pham *et al*.^2^ and Amde *et al*.^3^.

The applications of ILs are as diverse as their structures and the potential new anion-cation pairs are practically limitless. Surface-active ILs, i.e. ILs which decrease surface tension and interfacial tension, typically contain one or more long alkyl chains attached to the cation and/or anion head group. They have been used in numerous applications, such as, in extraction of organic compounds, metal ions and radioactive isotopes, as templates in production of porous materials, in microemulsions and as demulsifiers in petroleum chemistry (reviewed by Martínez-Palou and Aburto)^4^. In the present study novel surface-active cholinium- (Ch), guanidinium- (GND), and tetramethylguanidinium- (TMG) based ILs, in combination with isostearate, decanoate and neodecanoate anions, were investigated (Fig. 1 and Table 1). The ILs were developed as more advanced or low toxicity structures for enhanced extraction processes, to be used in biomass or petroleum chemistry, and the cation and anion pairs were selected from this application point of view.

When developing novel ILs for industrial applications, their toxicological effect is of importance due to the environmental responsibilities determined by legislation, such as, the European Union regulation regarding registration, evaluation, authorization and restriction of chemicals (REACH). Also, investigation of the IL structural properties and the toxicity mechanisms can provide useful information for the development of ILs that are efficient in their application, but less harmful for the environment.

Due to the structural variety of ILs in general, there are no mechanistic uniformity regarding the toxicity of ILs (reviewed by Egorova *et al*.)^5^. Based on the collective data, IL toxicity is dependent on certain structural properties of the monomeric cations and anions, such as the type of the ions, presence and nature of functional groups and the length of the attached alkyl chains (particularly for cations). However, the aggregation behaviour of the ILs needs to be taken into account as well. Considering the cation and anion species and the interactions between them, the biological activity and toxicity of substances may also be affected by micro-scale organization and aggregation of IL monomers.

The toxicity of the cation is commonly known to increase as a function of the alkyl chain length, as reviewed by Amde *et al*.^3^ There are only a few studies investigating the effect of the alkyl chains in the IL anions and particularly the impact of the carboxylate ILs. Based on these studies the elongation of the carboxylate alkyl chain increases the toxic effect in similar way as for cations^6^,^7^,^8^,^9^. However, this relationship between anion chain length and toxicity does not seem to be as widely recognized as the effect of the cation chain length, and therefore it needs to be further investigated.

In this study we investigated the acute cytotoxicities of the seven aforementioned ILs in aquatic environment. Due to the long-chain carboxylates the ILs in the present study were assumed to be surface-active and the nature of these anions were expected to determine the toxicities of the ILs. In contrast, the cations were expected to have a less significant role due to their short chain-length. The effect of the cations on the behaviour of the ILs was investigated by testing also the toxicities of the equivalent isostearate, decanoate and neodecanoate sodium salts, as reference materials. Isostearate and neodecanoate anions are derived from technical formulations of isostearic acid and neodecanoic acid. Both are branched acids. Isostearic acid is bio-based, derived from tall-oil fatty acid di/trimerisation and hydrogenation of the monomeric residue^10^. Neodecanoic acid is a low-cost petrochemical-based derivative, obtained through Reppe-Koch chemistry (acid-catalysed hydrocarboxylation) on octene isomers, derived from propene polymerisation^11^,^12^.

In the present study the toxicities were determined by exposing *Vibrio fischeri* marine bacteria to aqueous solutions of ILs at differing concentrations. These bacteria have successfully been used for IL toxicity assessments in a myriad of studies^7^,^13^,^14^,^15^,^16^,^17^,^18^,^19^,^20^,^21^,^22^,^23^,^24^,^25^,^26^,^27^,^28^,^29^,^30^,^31^,^32^,^33^,^34^,^35^,^36^,^37^,^38^,^39^. A recent comparison was performed between *V. fischeri* and three other standardised toxicity assays utilising aquatic organisms, namely *Daphnia magna* (a fresh water crustacean), *Selenastrum capricornutum* (a freshwater algae) and *Phaeodactylum tricornutum* (a seawater algae)^40^. Based on the study, *V. fischeri* was the most sensitive for the surface-active toxicants (detergents) and, therefore, these bacteria were selected for the toxicity assessment of the surface-active ILs in the present study.

The median effective concentrations (EC_50_) were determined for each IL. Since the toxic effect was thought to be caused by permeation of the surface-active molecules into the cell wall of the bacteria, related interactions of ILs with biomimetic liposomes were investigated utilizing differential scanning calorimetry (DSC). In DSC experiments, dipalmitoylphosphatidylcholine (DPPC) liposomes were used as biomimicking membranes and the change in the main phase transition temperature (T_m_) of the DPPC bilayer was followed, as a function of the IL concentrations. Such measurements could potentially offer more information on how the aforementioned ILs interact with the lipid bilayers. Lastly, the critical micelle concentrations (CMC) were defined for the ILs. Based on the CMCs, it can be defined whether the ILs are dissolved as singly dispersed molecules or if they form a dispersion of micelles, or other self-assembled structures (aggregates). Therefore, it can be determined if the interactions between liposomes take place between IL aggregates or singly dispersed IL molecules.

Our results demonstrate that the long chain carboxylate anions determine the toxicities of these ILs, whereas the non-surface-active cations have less impact on the toxicity. In addition, based on the DSC studies, the anion alkyl chain length and branching mainly define the extent of the interactions. However, in contrast to the toxicity studies, the anion impact is more affected by the accompanying cation.

## Results and Discussion

### Acute cytotoxicity

The toxicity measurements were performed mainly from an ecotoxicological point of view, since ILs are potentially hazardous for the environment and particularly for organisms in aquatic environments but also to humans. Therefore, *V. fischeri* marine bacteria were utilised in these measurements. EC_50_ values were determined for seven water-soluble surface-active ILs. The ILs in this study included Ch-, GND- and TMG-based ILs with straight and branched (iso and neo forms) fatty acid chains, namely isostearates, decanoates and neodecanoates. As a reference, the toxicities of the sodium salts of these carboxylates were measured in order to obtain information on the effect of the cation on the toxicities of the ILs.

**Figure 2:**
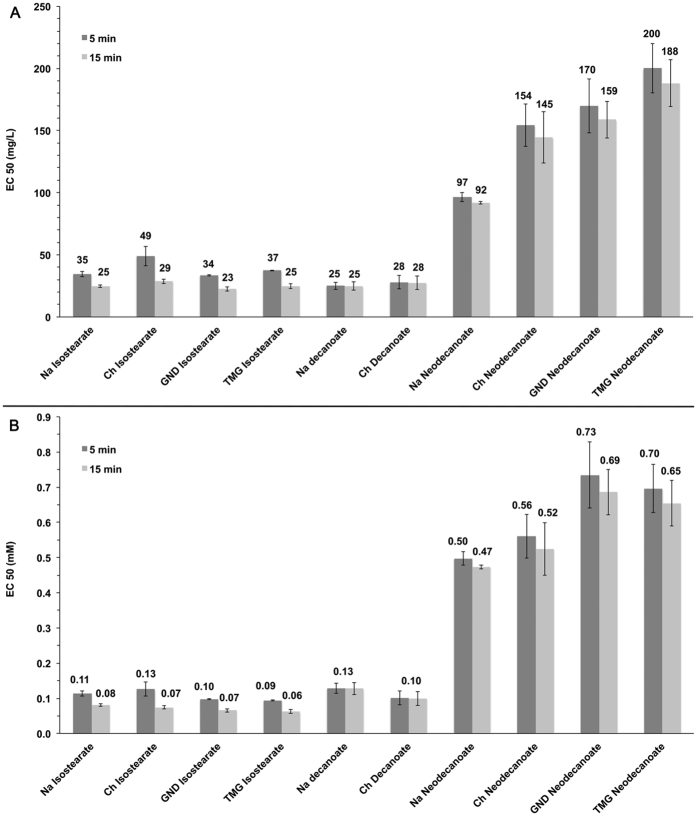
Toxicity of ILs. The EC_50_ values defined for the ILs using Vibrio Fischeri bacteria with 5 min and 15 min incubation. Presented as mass (**A**) and as molar (**B**) concentrations.

The current IL toxicity classifications are based on mass, not molar, concentrations. Therefore, the EC_50_ values are presented in Fig. 2A, based on the mass. As classified by Ruokonen *et al*.^8^ the ILs in this study were moderately toxic (decanoates and isostearates between 10–100 mg/L) or practically harmless (neodecanoates 100–1000 mg/L) according to their EC_50_ values. Two EC_50_ values, after 5 and 15 minutes incubation, are provided for reference purposes, since either of these incubation times is typically used in previous studies^7^,^13^,^14^,^15^,^16^,^17^,^18^,^19^,^20^,^21^,^22^,^23^,^24^,^25^,^26^,^27^,^28^,^29^,^30^,^31^,^32^,^33^,^34^,^35^,^36^,^37^,^38^,^39^ utilising *V. fischeri* bacteria. The toxic effect is time-dependent, particularly with relatively short incubation times (minutes time-scale). When the incubation time is increased, lower concentration is needed to achieve the median effect. Therefore 15-minute incubation results in higher toxicity (*i.e.* lower EC_50_ value).

Since the aim of the study was to investigate if the IL toxic properties are caused by the permeation of the IL into the lipid bilayer, the number of molecules potentially interacting with the bacteria is of most interest. The toxicities were normalised to the number of the molecules in the solution and, therefore, presented in Fig. 2B and in Table 1 as molar concentrations.

It is known from previous studies that longer alkyl chains increase the toxicity of ILs^3^,^7^,^8^,^9^,^13^,^15^,^18^. Therefore, merely based on chain lengths, the Na and Ch isostearates should be significantly more toxic than the corresponding decanoates (Fig. 2B). The similar toxicity of these isostearates and decanoates suggest that branching of the isostearates remarkably decreases their toxicity. Unfortunately, comparison with corresponding stearates was not possible due to their extremely poor water solubility. The difference between the EC_50_ values of Na and Ch decanoates and the corresponding neodecanoates evidently illustrate the effect of the branching on the toxicity: straight-chain decanoates are more toxic than the branched neodecanoates (average values ~0.1 mM vs. ~0.5 mM, see Fig. 2B for comparison).

Since the toxicity measurements were performed in 2% NaCl aqueous solution, subtle changes in the Na-cation concentrations did not affect the viability of the cells. Therefore, the choline cation did not have any practical effect on the toxicity of any of the Ch containing ILs: Ch isostearate and Ch neodecanoate are somewhat less toxic than their Na-cation-containing equivalents, whereas Ch decanoate is more toxic than Na decanoate (Fig. 2B). However, taking into account the standard deviations the differences are miniscule.

The GND and TMG isostearates have similar toxicities and appear to be somewhat more toxic than their Na and Ch equivalents (Fig. 2B). In contrast, within the neodecanoate group GND and TMG are evidently less toxic than their Na and Ch equivalents.

In summary, the length of the anion acyl chains seems to have a strong correlation with the toxicity of the ILs in this study. A similar effect has been observed before with differing ILs and test organisms. Based on Ruokonen *et al*.^8^, an increase in the anion alkyl chains of phosphonium-carboxylate ILs (C6, C10, C14, C16 and C18) increases the IL toxicity (measured using Chinese hamster ovary cell culture), as far as the molecules are dissolved as singly dispersed molecules^8^. Petkovic *et al*. showed that the increase in choline-carboxylate-IL toxicities follow the alkyl chain length (C2 < C3 < C4 < C5 < C6 < C8 < C10)^9^. Based on Rengstl *et al*. a similar effect was observed with choline-carboxylate ILs with C2-C10 using HeLa and SK-MEL28 cells (keratinocytes) – however, the trend was not linear^7^. Particularly, with shorter chains (<C6) there were large variations between the effects induced by the differing chain lengths. According to Muhammad *et al*., the toxicity of choline carboxylates measured with MCF-7 (a human breast cancer cell line) was actually slightly decreasing, as a function of the alkyl chain length (C2-C4 and C6)^6^. It seems that the length of the acyl chain mostly increases the toxicity with alkyl chain lengths > 6 carbons, whereas, with shorter chains the effects are not as predictable and may be more affected by the type of cation.

In the present study, branching of the anion alkyl chain decreased toxicity. In general, the effect of branching, however, is not as straightforward and seems to be greatly dependent on the head group of the surface-active component^9^,^27^,^31^,^41^.

Overall, in the present study the effect of the cation was small compared to the effect of the anion. Such an effect has also been detected in other studies, for instance, for choline carboxylates^7^,^9^. In the present study, the cation affected the EC_50_ values only within the neodecanoate analogues: surprisingly, GND and TMG cations decreased the toxicity of the ILs when compared to their Na and Ch equivalents.

### Differential Scanning Calorimetry

The aim of the DSC study was to investigate if there is a connection between the EC_50_ values defined with *V. fischeri* bacteria and the rupture point (or range) of the model lipid layer (DPPC). The ILs in the present study, and particularly their anionic components, are surface-active molecules. They are expected to permeate into DPPC liposome bilayers used as biomimetic membranes. This will result in a decrease in the order of the DPPC bilayers, and consequently in gradual transformation of the bilayer into a more fluidic phase, such as the ones formed by unsaturated phospholipids at room temperature. A similar approach in IL studies has been used by Gal *et al*.^42^ and Rengstl *et al*.^7^.

Since the T_m_ of pure DPPC layers is well established (approximately 41.3 °C) and very repeatable^43^, DPPC was chosen as the reference biomimetic membrane. The transition takes place when the lipid bilayer undergoes a transition from an ordered gel phase into a fluidic L-alpha phase. The phase transition of a lipid bilayer (such as DPPC) is a cooperative phenomenon (see more detailed description of cooperativity in Chiu and Prenner^44^), *i.e.* conformational change in one molecule forces adjacent molecules to adapt due to the high ordering of the bilayer. Therefore, phase transitions of bilayers made of pure lipids take place within a very narrow temperature range and results in sharp endothermic peaks. In contrast, impurities such as other surface-active molecules (surfactants) mixed with the phospholipids decrease the ordering of the bilayers. This decreases the cooperativity of the lipids and results in broader peaks.

Surfactants interact with or permeate into a lipid layer, the T_m_ starts to shift gradually towards lower temperatures, as a function of the surfactant concentration^43^. This is due to the decreased order of the lipids in the condensed gel-phase membrane. Due to the interactions between surfactants and liposomes, it is highly possible that new organised surfactant-lipid vesicles are formed. At least two differing scenarios are possible: i) The surfactants mixed with lipids disturb the organisation of the lipid layer, and the endotherm peak, caused by the transition, disappears gradually as the proportion of the surfactant increases. ii) The surfactant and the lipids form a new fairly organised bilayer, and formation of new phases is plausible. When the amount of the IL components in the bilayer grows high enough, the newly formed vesicle may also rupture (applies for both scenarios).

The mechanism of the rupture may be similar to what has been observed for neutral, anionic, and cationic surfactants and model lipid bilayers^45^,^46^. First the surface-active IL components permeate into the DPPC bilayer and IL/DPPC vesicles are formed (IL monomers + DPPC liposomes = IL/DPPC vesicles). At this phase an endotherm produced by the main phase transition is observed, but at a different T_m_ than for the pure lipid layer. The permeation of the surfactant takes place until the membrane is saturated with the surfactant. When the concentration of the surfactant is increased beyond a certain IL- and/or lipid-dependent threshold, it is plausible that the bilayer gradually disintegrates and additional IL/DPPC aggregates are formed. Therefore, possibly a state is reached in which aggregates coexist with the remaining IL/DPPC vesicles. However, the aggregates may not possess such an organised structure that any phase transitions would take place. Endotherm would only be produced by the transition originating from the remaining IL/DPPC bilayers. When the concentration of the surfactant is further increased, the IL/DPPC vesicles rupture and remain as IL/DPPC aggregates.

**Figure 3:**
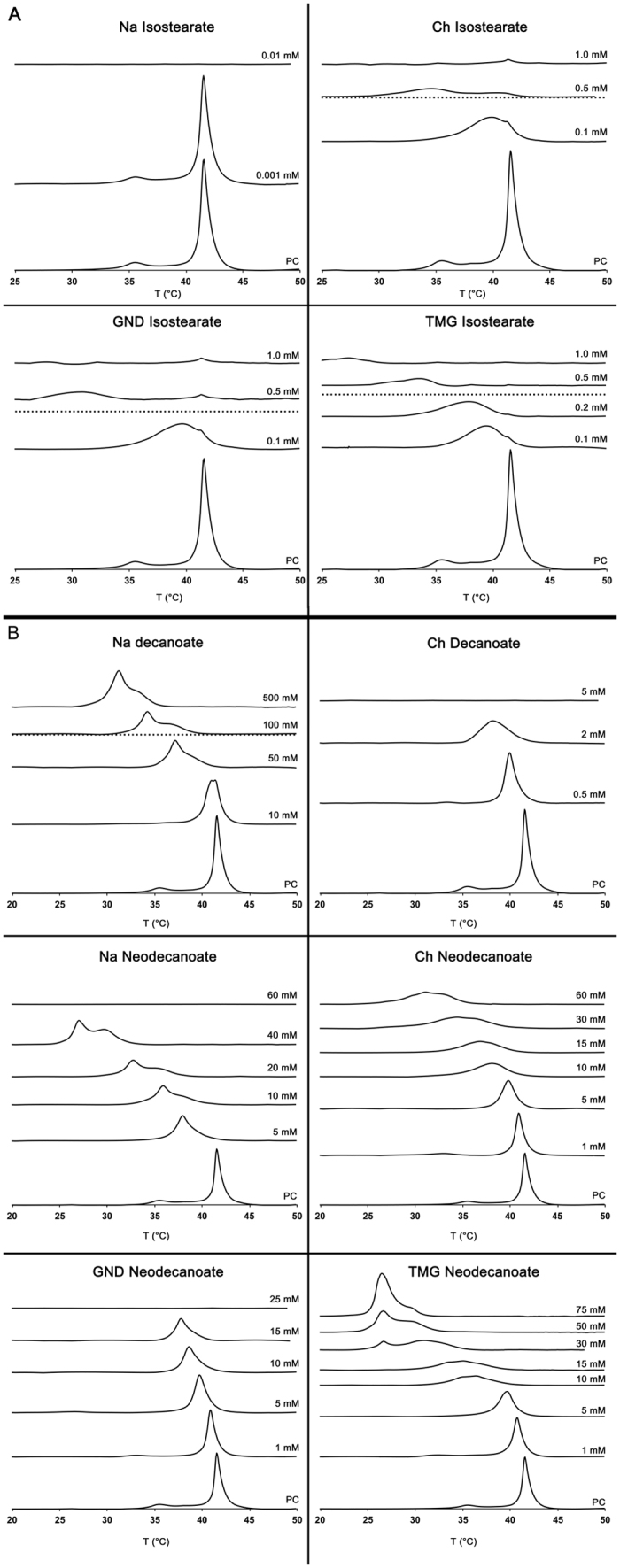
Interactions of ILs with DPPC liposomes described by DSC. Thermograms measured for pure DPPC liposomes (0.4 mM) and mixtures of DPPC liposomes and differing concentrations of isostearates (**A**), decanoates and neodecanoates IL (**B**). The peaks represent endothermic phase transitions. The broken lines (where applicable) designate the limit after which the ILs are dispersed as aggregates (CMC).

The DSC experiments were performed in order to determine the IL concentrations that induce a shift in the DPPC bilayer phase transition temperature. Figure 3 illustrates the representative thermograms, which were recorded during the second heating scans. Since the heating and the cooling scans were performed three times, the second heating thermogram was selected as the representative one. Often the first thermogram differed from the two latter ones, which mostly were identical to each other. If the baseline variations observed during the first heating scans were not observed on the following two scans and with other concentrations of the same ILs, they were regarded as artefacts. With certain ILs, the intensity of the peaks was decreasing with the heating scan. Slight shifting of the peaks was also observed. These changes are most likely due to reorganisation of the IL-lipid membranes as the system is heated up and cooled down. The difference observed particularly between the first and the second heating scans is explained by the fact that; when the DPPC lipid layer is heated to a fluidic state, the IL components can more easily permeate into the lipid layer and the system equilibrates. During the cooling and a second heating scan the surface-active impurities are already within the layer and therefore, less change is observed between the two latter scans. Cooling scans (not shown) were recorded in order to follow the stability of the system, however, no further analysis of the cooling scans was performed. Such heating and cooling scans are standard in DSC experiments.

The EC_50_ values provided by the toxicity assays may be dependent on differing complex mechanisms of interaction between the toxicant and the organism. In contrast, the interactions between the surface-active toxicant and liposomes are simpler. The concentration of the IL generating an observable impact on the bilayer phase transition (effective concentration) is dependent, not only on the concentration and properties of the ILs, but also on the concentration of the liposomes (molar concentration of lipid). The effect is dependent on the ratio of these two components. Therefore, due to the differences between the two systems, the effective concentrations of the ILs obtained form DSC were not expected to be similar to the EC_50_ values. The 0.4 mM lipid concentration was chosen since it gives a symmetrically shaped and repeatable signal with the calorimeter that we used.

The DSC results are discussed in more detail in the following text for each IL carboxylate group. The concentration range within which the phase transition endotherm was lost is presented in Table 1. Instead of referring to the peak areas, which define the energy taken by the transition (cal/mol), corresponding peak heights are referred to in the text, since the relative changes in the peak heights are more easily observed by visual inspection.

#### Isostearates

The behaviour of Ch, GND and TMG isostearates were very similar and largely independent of the cation (Fig. 3A). Already 0.1 mM concentrations caused the transitions to considerably shift to a lower temperature and the peak lost its sharp profile, suggesting that the order and cooperativity within the bilayer was remarkably decreased. At 0.5 mM the transitions shifted to ~35 °C and below, depending on the IL, and the peak height further decreased from the previous concentrations. The peaks caused by the main phase transitions were not detected anymore at 1.0 mM concentrations – a faint broad peak was still only barely visible for TMG isostearate.

Interestingly, a faint endotherm at the original DPPC transition temperature remained throughout the concentration range, particularly noticeable in Ch and GND isostearate thermograms. For GND isostearate 5, 10 and 20 mM concentrations were also measured (not shown in Fig. 3A) and the small peak was still observable in all of these thermograms, similar to the 0.5 and 1.0 mM concentrations. Also with TMG a barely detectable signal was observed in the first heating scan thermograms at 0.5 mM and 1.0 mM concentrations, but the peak was not visible in the second and third scans, as shown in Fig 3A. In all of these experiments the intensity of these small peaks decreased approximately 2–4 fold between the first and second heating scans. It seems that for some reason, either a small amount of DPPC liposomes remain unaffected in the solution or phase separated regions remain in the IL/DPPC vesicles.

The behaviour of Na isostearate deviated substantially from the other isostearates since the loss of the main phase transition, probably caused by the rupture of the bilayer, takes place already between 0.01 and 0.001 mM concentration. This collective data suggests that with isostearates the cation also has an impact on the IL-liposome interactions considerably decreasing the harmful effect of the anion.

#### Decanoates

The decanoates, having very similar EC_50_ values as the isostearates, show somewhat differing behaviour in the DSC experiments (Fig. 3B). Compared to isostearates, considerably higher Na decanoate concentrations were needed to induce a shift in T_m_. Minor shift in T_m_ was visible at 10 mM concentration. Closer observation implies that the peak is formed by two overlapping endotherms, suggesting that two overlapping endothermic transitions take place. In addition to gradual shit in T_m_, the peak height further decreased at 50 and 100 mM concentration but increased at 500 mM concentration. In addition, with increasing decanoate concentration the overlapping endotherms became more evident. These observations suggest that new mixed IL-lipid vesicles are formed and that the bilayer can adapt a somewhat organised phase, when a certain IL-lipid proportion is reached. However, detailed phase transition behaviour of these newly formed vesicles is out of the scope of this study. Compared to Na decanoate, remarkably lower concentrations were needed for Ch decanoate. The loss of the transition took place between 2 to 5 mM concentration, possibly due to the rupture of the vesicle. Comparison of the data obtained for Na and Ch decanoates, suggests that the presence of the choline cation remarkably modifies the effect of the decanoate anion.

Rengstl *et al*. investigated the effect of the Ch nonanoate (C9) on DPPC liposomes using DSC^7^. Surprisingly, the effective concentrations were remarkably larger than the ones observed here with Ch decanoate. The concentration was increased up to 50 mM and a peak for the T_m_ was still observed, whereas the endotherm was lost between 2 and 5 mM concentration using Ch decanoate in the present study.

#### Neodecanoates

The behaviour of Na neodecanoate was similar to that of Na decanoate, however, at considerably lower concentrations (Fig. 3B). Another transition is visible at 10 mM concentration and is more evident at 20 mM and 40 mM concentrations. In contrast to Na decanoate, the phase transition was lost between 40 mM and 60 mM concentrations most likely due to the rupture of the liposome. Ch and GND neodecanoates showed a more predictable behaviour. For Ch neodecanoate a faint broad peak caused by the transition was observed until 60 mM concentration. Since no significant change in the peak height was observed between 10 mM and 60 mM concentrations, no higher concentrations were measured. GND neodecanoate showed very predictable and consistent behaviour until the rupture of the vesicle between 15 and 25 mM concentration.

TMG neodecanoate showed very similar behaviour to GND neodecanoate until a concentration of 5 mM. At 15 mM, only a very faint broad peak was observed. Increasing the concentration further, up to 30 mM, resulted in another transition. The intensity of this peak increased with increasing concentration, outgrowing the intensity of the original transition. 75 mM pure TMG neodecanoate was also measured as a reference and only a very minor signal (not shown) barely deviating from the baseline was observed, at approximately the same transition temperature as the largest peak in the 75 mM IL/DPPC experiment. This data also suggests that a new phase is formed when a certain IL/lipid proportion is reached within the vesicle bilayer. This is supported by the results obtained with fatty acid/DPPC vesicles by changing the fatty acid proportion within the bilayer^47^. Similar formation of new endothermic peaks, below the T_m_, were detected with oleic acid. According to the authors, these are caused by formation of coexisting phases in the vesicles.

### Critical micelle concentrations

The critical micelle concentrations were measured in order to define the concentration threshold for aggregation of singly dispersed IL molecules (micelle formation). The ILs may interact differently with liposomes depending on whether they are as singly dispersed molecules (monomers) or as aggregates. Since the IL anions in this study are surface-active isostearates, decanoates and neodecanoates, the anion is expected to mainly define the CMC of the IL.

**Figure 4:**
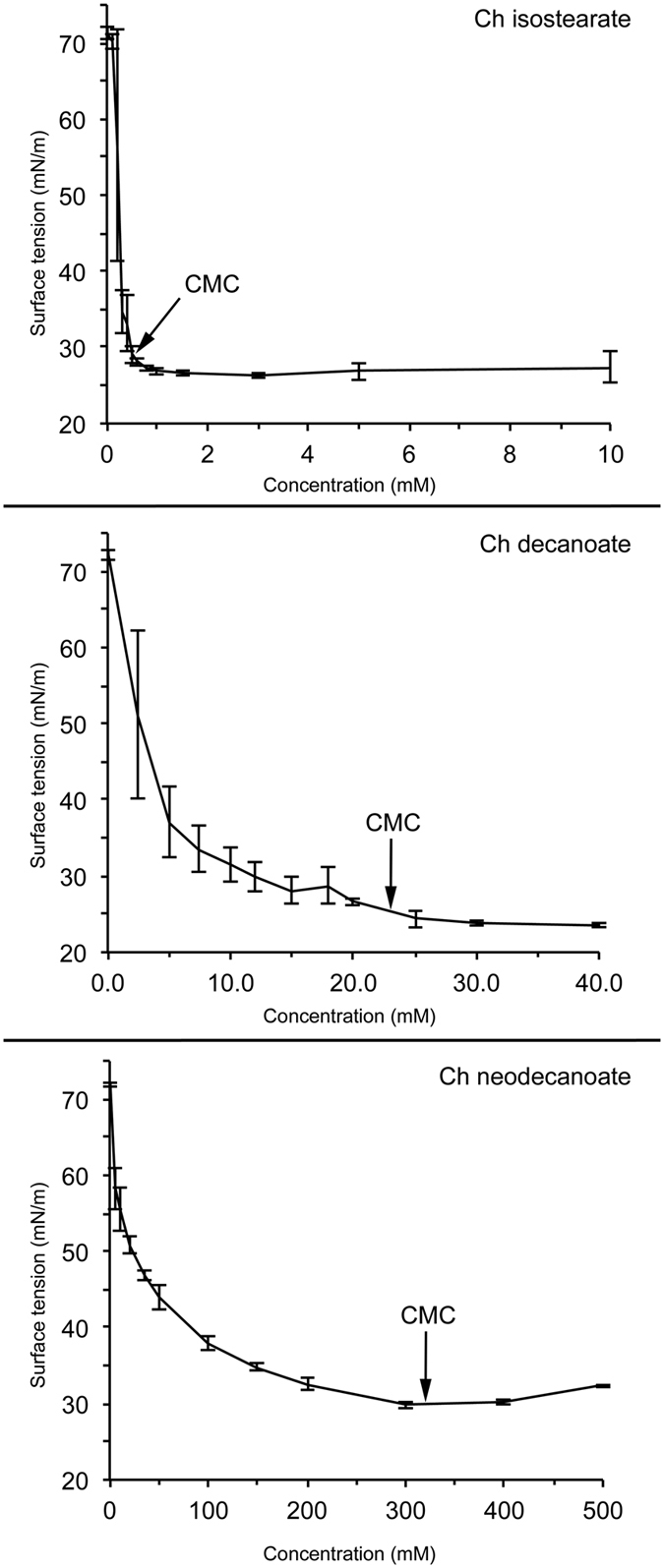
CMC-determination graphs for Ch isostearate, Ch decanoate and Ch neodecanoate. Graphs exemplify the differing surfactant properties of each IL. The surface tension data obtained using the pendant drop method (see ‘Methods’ for more detailed description) was plotted as a function of the IL concentrations. The exact CMCs were defined by logarithmic fitting (see the Results and Discussion for more detailed description).

The CMCs for the ILs are presented in Table 1. As expected, for long-chain isostearates, CMCs are lower than for shorter-chain decanoates. Also, branching of the neodecanoates increases the CMC. Figure 4 exemplifies three differing graphs used for defining the CMCs of Ch isostearate, decanoate and neodecanoate. For Ch isostearate, a distinct break point is noticeable at a concentration of ~0.5 mM. For such cases, linear, sigmoidal and logarithmic fits give very similar results independent of the fitting method. For Ch decanoate and neodecanoate, the slope is milder and the determination of a specific point for the CMC is more difficult. For such ILs the logarithm of the IL concentration and logarithmic fit was utilised since more precise values can be obtained using this method. The fitting method used for each IL is designated in Table 1. A logarithmic fitting was used for all ILs, except for GND and TMG isostearate for which a sigmoidal fitting was utilised.

Among the isostearates, the average CMC values are in the same order of magnitude, i.e. 0.3 – 0.7 mM, demonstrating that the presence of the cation does not remarkably affect their CMCs. The CMCs for the decanoates, 90 ± 3 mM for Na and 23 ± 4 mM for Ch decanoate differ considerably. The CMC for Na decanoate is in line with widely accepted value of ~100 mM^48^. Interestingly the CMC for Ch decanoate is somewhat lower than the value of 75 mM determined in a previous study^7^. For neodecanoates, the average values are notably higher – roughly between 100 and 450 mM. The cation seems to affect the CMCs of all of the ILs in this study. However, the effect is IL-dependent.

Considering the potential effect of aggregation on toxicity, in theory the EC_50_ values determined for isostearates could be affected by IL aggregation. Aggregation of ILs does not necessarily take place at a sharply defined concentration (i.e. precisely at the CMC) and, hence, IL components begin to aggregate before the CMC is reached. This pre-CMC aggregation decreases the number of free (potentially toxic) monomers in the solution and therefore may decrease the IL toxicity. Since the isostearates have CMCs close to their EC_50_ values, such aggregation could explain why the apparent toxicity of isostearates is not as high as the anion chain length would suggest, for instance when compared to decanoates. However, there is an evident break point detected in the isostearate CMC plots (see Fig. 4 for Ch decanoate plot). This suggests that for the isostearates the aggregation takes place rapidly very close to the CMC i.e. no aggregation takes place below the CMC. Therefore, the practical effect of pre-CMC aggregation for the isostearates is not considerable. Since the CMCs of decanoates and neodecanoates are considerably higher than their EC_50_ values, their aggregation does not affect their toxicity determination either. The impact of CMCs on DSC results is discussed in more detail below.

#### The effect of CMCs on ionic liquid-liposome interactions

In DSC experiments, the aggregation of the ILs may affect the interactions between the ILs and the liposomes. With Ch, GND and TMG isostearate, the effective concentrations are very close to their CMC values (Table 1). For Ch, GND and TMG isostearate an endotherm is observed at concentrations just below the CMC (at 0.1 mM for Ch, GND and at 0.2 mM for TMG isostearate, see Fig. 3A). However, the endotherm is barely visible at 0.5 mM concentration and at 1 mM concentration no peak is visible. Further, no endotherms were observed for 2 mM Ch, GND and TMG isostearates (thermograms not shown). This data suggests that aggregation has no impact on the IL-liposome interactions. There are at least two possible explanations for these observations.

i) One possible mechanism for such behaviour is presented in the following; When the IL concentration in a mixture of ILs and DPPC liposomes is below the CMC, the free surface-active IL monomers permeate into the DPPC bilayer and most likely remain there. When the concentrations of the ILs and phospholipids are in the same order of magnitude (as with isostearates), a state is reached where all the IL monomers are incorporated into the bilayer and none exist free in the solution. Alternatively, the bilayer is saturated with the ILs, the monomers stop permeating into the bilayer, and a certain concentration of monomers remain in the solution. This is very likely when the concentration of the IL greatly exceeds the concentration of the lipids. In contrast, above the CMC there is a coexistence of IL monomers and IL aggregates. At and above the CMC the concentration of the monomers equals the CMC and all the monomers above this concentration are included in aggregates. Similar to the description above, free monomers can still permeate into the liposomes. Since the concentration of free IL monomers in the solution is decreasing, IL aggregates gradually disintegrate into free monomers due to the dynamic equilibrium existing between free monomers and aggregates. This aggregate disintegration process takes place until the concentration of the monomers again equals the CMC, or when there are no IL aggregates remaining. Therefore, with this mechanism the apparent impact of aggregated ILs can be similar to that of free monomers.

ii) Another possible explanation is the effect of temperature on the CMCs. The CMCs of anionic surfactants are known to increase somewhat as a function of temperature^49^. Therefore, a similar effect could also be caused by an increase in the CMCs during the heating scans. First, when the CMC is exceeded at lower temperature (e.g. at room temperature), the ILs form aggregates. When the temperature is raised during the heating scans, the aggregates disintegrate into monomers due to the consequential raise in the CMC. Therefore, more free monomers are available to interact with the DPPC liposomes.

For Na isostearate no endotherm is observed at 0.01 mM concentration. Since this concentration is considerably lower than the CMC, singly dispersed IL molecules interact with the liposomes. The loss of the endotherm is caused either by formation of a new fluidic IL-lipid phase or by rupture of the membrane.

The IL concentration range during the DSC experiments was clearly below the CMCs for the decanoates and neodecanoates, except for Na decanoate, which was used as a reference for the ILs. Therefore, excluding Na decanoate, the data clearly shows that singly dispersed IL molecules interact with the liposomes. Since the CMC of Na decanoate is ~90 mM, aggregates are present at 100 mM and 500 mM concentrations. The mechanism of interaction is possibly based on similar aggregate disintegration as described above for isostearates. A thermogram was measured for pure 250 mM sodium decanoate and no transitions were observed. This suggests that the endotherms actually result from the transition taking place in the decanoate/DPPC vesicles.

## Summary

Seven Ch-, GND- and TMG-based ILs with long chain anions were investigated. The toxicities were defined mainly by the long chain anions – the longer the chain, the more toxic the compound. The branching of the anions decreased the toxicities of the ILs. The cations did not have a significant impact on the toxicities of decananoates and isostearates, but the GND and TMG neodecanoates were surprisingly somewhat less toxic than their Ch equivalent.

DSC experiments were utilised to investigate the potential effect of the surface-active ILs on DPPC liposomes. These liposomes were used as biomimetic lipid bilayers and the T_m_ of the membrane was followed as a function of the IL concentration. The chain length of the anion determined greatly the extent to which the ILs affected the liposomes, as well as the toxicities. The isostearates affected the liposomes at concentrations < 1.0 mM, Ch decanoate at concentrations of 0.5–5 mM, and the neodecanoates at concentrations of ≫1 mM. The presence of Ch, GND or TMG cations evidently had an impact on the behaviour of all the anions, when compared with the Na salts of each anion. There were no considerable differences between Ch, GND and TMG isostearates, whereas, differing effects induced by the cations were observed for decanoates and neodecanoates. However, no trend was observed for the effect of the cations, moreover, the behaviour was IL-dependent.

Based on this study, DSC is a valid tool for investigating the interactions between the surface-active ILs and biomimetic lipid layers. According to the present study tentative predictions of the toxicity of comparable (similar types of) ILs can be done. However, the toxic IL concentrations cannot be estimated. Considering bacterial cell walls or other biological membranes and the biomimetic DPPC liposomes used in this study, two very different lipid layers are investigated. The DPPC forms highly organised bilayers below its T_m_, whereas the bacterial cell walls, as well as other biological lipid bilayers, are mainly fluid phase mixtures of several differing lipid species and proteins. Therefore, the fluid lipid layers are more susceptible for permeation of surface-active impurities than the condensed DPPC bilayers. However, the bacteria may have protective mechanisms against toxicants, and also the mechanism of toxicity may be highly complex. Therefore, it cannot be expected that the EC_50_ concentrations would match the effective concentrations used in the DSC experiments.

Also, analysis of DSC results is dependent on what is considered to be a toxic concentration in the model membrane. A very small proportion of impurities permeating into the membranes of the model organism may be toxic. In contrast, a similar proportion of lipids in the biomimetic membranes may not have any considerable effect on the phase transition behaviour. In this study the aim was to investigate large-scale effects such as the rupture of the liposomes. Therefore, the effective concentrations of the ILs are high compared to the EC_50_ values, particularly for shorter chain decanoates and neodecanoates.

Consequently, it needs to be remembered that even though the toxicities of the ILs in this study depended on their surface-active properties, the damage that they cause by permeating into the model organism bilayer may not be structural in essence. Moreover, the ILs may interfere with the function of vital membrane-dependent cell signalling pathways by disturbing the subtle organisation of lipids and proteins in the bilayer.

## Methods

### Chemicals and reagents for toxicity and interaction studies

DPPC was purchased from Avanti Polar Lipids (Alabaster, AL, USA). The HPLC grade chloroform was acquired from WVR international (Leuven, Belgium). The bacteria and the chemicals for running the Microtox assay where purchased form Modern Water (New Castle, DE, USA). The ultrapure water used in the experiments was first distilled and then filtered, and deionized using Milli-Q device.

### Synthesis of ionic liquids

All ILs were prepared by simple mixing of the reagents in presence or absence of solvents, i.e. the base with the respective acid in a 1:1 molar ratio. Some reagents required elevated temperature or solvents; i.e, NaOH (pellets) and (solid) decanoic acid were pre-dissolved in methanol. Crystalline guanidinium carbonate required reaction in methanol at 50 °C. Methanol was removed after acid-base synthesis by rotary evaporation down to ~10 mbars. Rotary evaporation was also employed to remove water, where present. Neodecanoic acid (Versatic™ acid 10) was kindly provided by Hexion Speciality Chemicals (Beveren, Belgium). Isostearic acid was the brand Pristorine™ 3501 (Croda International Plc, Snaith, East Yorkshire, UK), derived from di/trimerisation and hydrogenation of tall oil fatty acid grades. Detailed characterization-assignment of functional groups was performed by ^1^H and ^13^C NMR (600 MHz) in D_2_O or DMSO-d_6_. Karl-Fischer titration was used to monitor water contents to below 1 wt%. All structures were either glass, liquid or solid at room temperature. Differential scanning calorimetry (DSC) and thermogravimetric analysis (TGA) data will be presented in further publications.

Four procedures were used to synthesise the different cation classes of ionic liquid (choline, guanidnium, 1,1,3,3-tetramethylguanidinium or sodium):

Choline isostearate (example): Isostearic acid (317 g) was added in one portion to a commercial solution of choline hydroxide (45%wt. in methanol, 300 mL, 281 g) in a 1 L Erlenmeyer flask. The solution was mixed at room temperature, upon which a mild exotherm was observed. Mixing was continued for a further 30 min. The product was then rotary evaporated in batches to remove methanol. A soft gel was formed which was left in the flask to cool overnight. The dry fractions were combined and bottled the following day to give quantitative yield. NMR analysis showed only trace impurities (Supplementary Information).

Guanidinium isostearate (example): Isostearic acid (370 g) was added in one portion to a solution of guanidinium carbonate (117 g) in methanol (217 g), in a 1 L Erlenmeyer flask. This was mixed for 45 min at 50 °C until a clear solution was obtained. The product was dried by rotary evaporation in batches. A soft gel was formed which was left in the flask to cool overnight. The dry fractions were combined and bottled the following day to give quantitative yield. NMR analysis showed only trace impurities (Supplementary Information).

1,1,3,3-Tetramethylguanidinium isostearate (example): TMG (145 g) was added over the space of a few minutes to isostearic acid (355 g) in 1 L Erlenmeyer flask. A mild exotherm was observed. The mixture was left to cool down to produce a soft gel in quantitative yield. NMR analysis showed only trace impurities (Supplementary Information).

Na isostearate (example): Crushed Na hydroxide pellets (1.22 g) were added over the space of a few minutes to isostearic acid (8.65 g) in methanol (12.5 g) in a 100 mL Erlenmeyer flask. A mild exotherm was observed. The mixture was left to cool down to produce a soft gel in quantitative yield. NMR analysis showed only trace impurities (Supplementary Information).

### Preparation of liposomes

A stock solution of DPPC was prepared by dissolving DPPC in chloroform. Required amount of the stock solution was transferred into a glass test tube and the chloroform was evaporated under airflow in order to form a thin film of DPPC on the walls of the tube. The air-dried lipid film was held under vacuum desiccator at least for 2 h to remove the residual solvent. The lipid was dispersed into water to obtain a 4.0 mM liposome stock. The dispersion was incubated in an ultrasonication water bath for 30 min at 60 °C facilitating the formation of multilamellar liposomes.

### Vibrio fischeri toxicity assay

The toxicities of the ILs were defined by measuring their EC_50_ values using *V. fischeri* bacteria and Microtox luminometer/thermostate apparatus (Modern Water, USA). The response measured in this assay is the decay of bioluminescence produced by the bacteria. In short, the bioluminescence was measured before the exposure on the potentially toxic compound to determine the baseline luminescence. Consequently, the bacteria were exposed on minimum four differing toxicant concentrations in 2% (w/v) NaCl solution and the decay in the bioluminescence was recorded. Based on the concentration-dependent decay the EC_50_ value was defined. The toxicities were defined at set time intervals of 5 and 15 minutes. Two independent measurements were performed for each IL as duplicates.

### Differential scanning calorimetry

DSC was utilised in observing the changes induced by the ILs in the organisation of the lipid bilayers. DPPC was used because the T_m_ of the bilayer is well recognised – approximately 41.3 °C^43^. Typically, when surface-active compounds permeate into the lipid layer, the transition temperature starts to shift with an increasing proportion of the compound in the lipid layer^47^. There are two additional transitions observed for DPPC aqueous solutions; the subtransition at 21 °C and the pretransition at 36 °C. However, the subtransition at 21 °C is visible only if the lipid is incubated below the transition temperature for several days. The transition at 36 °C is highly dependent on the heating rate and therefore may have somewhat differing values depending on the experimental setup^43^.

DSC experiments were performed using a VP-DSC MicroCalorimeter (MicroCal LLC, MA, USA). Aqueous stocks of DPPC liposome dispersion and IL solution were mixed resulting in a lipid concentration of 0.4 mM with varying concentration of IL. The samples were degassed under vacuum in order to avoid bubble formation during the sample loading and the heating/cooling scans. The mixtures were exposed to a heating scan starting from 15 °C and ending at 50 °C and subsequent cooling scan from 50 °C to 15 °C. The heating/cooling scans were repeated three times for each sample. The temperature was allowed to stabilise for 30 min before each heating scan. The temperature ramp was 60 °C/hour. The reference during the measurement was ultrapure water.

### Critical micelle concentrations

Surfactants dissolve in aqueous solutions as monomers to certain concentration threshold, after which they start to build up into differing aggregates. This concentration threshold is called critical micellar concentration (CMC). However, for many surfactants the CMC is moreover a concentration range, not a specific concentration point, and the width of this CMC region depends greatly on the structure of the compound. In its simplest form the surfactants, such as fatty acids, form micelles, as the term critical micellar concentration suggests. Other surfactants such as phospholipids, however, form for instance liposomes, regardless, the term CMC is used. CMC and the form of the aggregates are dependent on the surfactant structure.

The optical pendant drop method using a contact angle meter (CAM 200, KSV Instruments, Espoo, Finland) was used for determining the CMCs. In short, a series of differing IL concentrations in water solution was investigated. A pendant-drop of a constant height was formed and the surface tension of the drop was determined based on the drop curvature by using a fitting method according to Young-Laplace equation. The surface tension was plotted as a function of the IL concentration.

The surface tension of the drop decreases as a function of increasing surfactant concentration because the singly dispersed IL molecules diffuse and orientate at the air-water interface and therefore reduce the surface tension. When the concentration reaches the CMC, additional molecules above this concentration form aggregates instead of diffusing to the surface of the drop. Therefore no change in surface tension is observed.

The CMCs were determined based on the intersection point of two trend lines fitted based on the data. The surface tension was either plotted as a function of IL concentration or logarithm of the concentration and either sigmoidal or logarithmic fit, respectively, was used. The choice of method was dependent on which method provided the best fit.

## Additional Information

**How to cite this article:** Rantamäki, A. H. *et al*. Impact of Surface-Active Guanidinium-, Tetramethylguanidinium-, and Cholinium-Based Ionic Liquids on *Vibrio Fischeri* Cells and Dipalmitoylphosphatidylcholine Liposomes. *Sci. Rep.* **7**, 46673; doi: 10.1038/srep46673 (2017).

**Publisher's note:** Springer Nature remains neutral with regard to jurisdictional claims in published maps and institutional affiliations.

## Supplementary Material

Supplementary Information

## Figures and Tables

**Figure 1:**
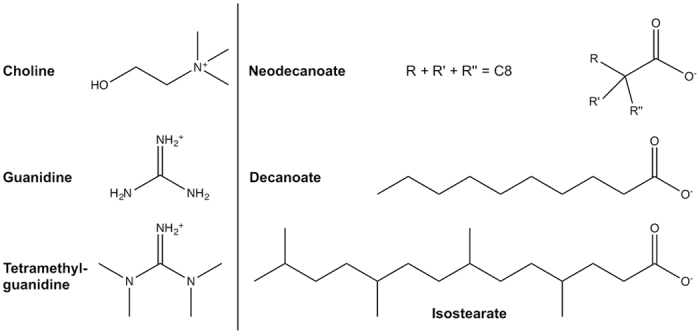
Structures of the IL cations and anions. Neodecanoate and isostearate technical fomulations are mixtures of isomers. The structures shown are examples.

**Table 1:** The properties measured for the ILs: the EC_50_ values were determined using *Vibrio Fischeri* bacteria with 5 min and 15 min incubation; the concentration range within which the phase transition was no longer detected (Loss of transition); the critical micelle concentration (CMC) and the fitting method used for the CMC determination (superscript).

Ionic liquid	M (g/mol)	EC50_5min_ (mM)	EC50_15min_ (mM)	Loss of transition (mM)	CMC (mM)
Na Isostearate	306.47	0.11 ± 0.01	0.081 ± 0.004	0.001–0.01	0.72 ± 0.06^L^
Ch Isostearate	387.65	0.13 ± 0.02	0.074 ± 0.005	0.5–1.0	0.50 ± 0.06^L^
GND Isostearate	343.56	0.098 ± 0.002	0.066 ± 0.005	0.5–1.0	0.31 ± 0.08^S^
TMG Isostearate	399.66	0.094 ± 0.001	0.062 ± 0.005	0.5–1.0	0.30 ± 0.04^S^
Na decanoate	194.24	0.13 ± 0.02	0.13 ± 0.02	—	90 ± 3^L^
Ch Decanoate	275.43	0.10 ± 0.02	0.10 ± 0.02	2.0–5.0	23 ± 4^L^
Na Neodecanoate	194.24	0.50 ± 0.02	0.473 ± 0.006	40–60	422 ± 38^L^
Ch Neodecanoate	275.43	0.56 ± 0.06	0.52 ± 0.08	—	319 ± 13^L^
GND Neodecanoate	231.34	0.73 ± 0.09	0.69 ± 0.07	15.0–25.0	118 ± 2^L^
TMG Neodecanoate	287.45	0.70 ± 0.07	0.65 ± 0.07	—	261 ± 11^L^

L Logarithmic fitting.

S Sigmoidal fitting.

## References

[b1] Cvjetko Bubalo M., Radošević K., Radojčić Redovniković I., Halambek J. & Gaurina Srček V. A brief overview of the potential environmental hazards of ionic liquids. Ecotox. Environ. Safe. 99, 1–12 (2014). 10.1016/j.ecoenv.2013.10.019 24210364

[b2] Thuy Pham T. P., Cho C.-W. & Yun Y.-S. Environmental fate and toxicity of ionic liquids: A review. Water Res. 44, 352–372 (2010). 19854462 10.1016/j.watres.2009.09.030

[b3] Amde M., Liu J.-F. & Pang L. Environmental application, fate, effects, and concerns of ionic liquids: A review. Environ. Sci. Technol. 49, 12611–12627 (2015). 26445034 10.1021/acs.est.5b03123

[b4] Martínez-Palou R. & Aburto J. Ionic liquids as surfactants–applications as demulsifiers of petroleum emulsions In Ionic liquids - current state of the art (ed. Handy S.) 305–326 (InTech, 2015).

[b5] Egorova K. S., Gordeev E. G. & Ananikov V. P. Biological activity of ionic liquids and their application in pharmaceutics and medicine. Chem. Rev. 10.1021/acs.chemrev.6b00562 (2017). 28125212

[b6] Muhammad N. . Synthesis and physical properties of choline carboxylate ionic liquids. J. Chem. Eng. Data 57, 2191–2196 (2012).

[b7] Rengstl D., Kraus B., Van Vorst M., Elliott G. D. & Kunz W. Effect of choline carboxylate ionic liquids on biological membranes. Colloids Surf B Biointerfaces 123, 575–581 (2014). 25444662 10.1016/j.colsurfb.2014.09.057 PMC4509629

[b8] Ruokonen S.-K. . Effect of ionic liquids on zebrafish (danio rerio) viability, behavior, and histology; correlation between toxicity and ionic liquid aggregation. Environ. Sci. Technol. 50, 7116–7125 (2016). 27253865 10.1021/acs.est.5b06107

[b9] Petkovic M. . Novel biocompatible cholinium-based ionic liquids-toxicity and biodegradability. Green Chem. 12, 643–649 (2010).

[b10] Anneken D. J. . Fatty acids In Ullmann’s encyclopedia of industrial chemistry (Wiley-VCH Verlag GmbH & Co. KGaA, 2000).

[b11] Fefer M. Neo acids: Synthetic highly branched organic acids. J. Am. Oil Chem. Soc. 55, A342–A348 (1978).

[b12] Kubitschke J., Lange H. & Strutz H. Carboxylic acids, aliphatic in Ullmann’s encyclopedia of industrial chemistry (Wiley-VCH Verlag GmbH & Co. KGaA, 2000).

[b13] Ranke J. . Biological effects of imidazolium ionic liquids with varying chain lengths in acute vibrio fischeri and wst-1 cell viability assays. Ecotox. Environ. Safe. 58, 396–404 (2004). 10.1016/S0147-6513(03)00105-2 15223265

[b14] Docherty K. M. & Kulpa C. F. Jr Toxicity and antimicrobial activity of imidazolium and pyridinium ionic liquids. Green Chem. 7, 185–189 (2005).

[b15] Matzke M. . The influence of anion species on the toxicity of 1-alkyl-3-methylimidazolium ionic liquids observed in an (eco) toxicological test battery. Green Chem. 9, 1198–1207 (2007).

[b16] Samori C., Pasteris A., Galletti P. & Tagliavini E. Acute toxicity of oxygenated and nonoxygenated imidazolium-based ionic liquids to daphnia magna and vibrio fischeri. Environ. Toxicol. Chem. 26, 2379–2382 (2007). 17941742 10.1897/07-066R2.1

[b17] Stolte S. . Effects of different head groups and functionalised side chains on the aquatic toxicity of ionic liquids. Green Chem. 9, 1170–1179 (2007).

[b18] Romero A., Santos A., Tojo J. & Rodriguez A. Toxicity and biodegradability of imidazolium ionic liquids. J. Hazard. Mater. 151, 268–273 (2008). 18063302 10.1016/j.jhazmat.2007.10.079

[b19] Ventura S. P. M., Goncalves A. M. M., Goncalves F. & Coutinho J. A. P. Assessing the toxicity on c(3)mim tf2n to aquatic organisms of different trophic levels. Aquat. Toxicol. 96, 290–297 (2010). 20018392 10.1016/j.aquatox.2009.11.008

[b20] Samorì C. . Introduction of oxygenated side chain into imidazolium ionic liquids: Evaluation of the effects at different biological organization levels. Ecotox. Environ. Safe. 73, 1456–1464 (2010). 10.1016/j.ecoenv.2010.07.020 20674022

[b21] Alvarez-Guerra M. & Irabien A. Design of ionic liquids: An ecotoxicity (vibrio fischeri) discrimination approach. Green Chem. 13, 1507–1516 (2011).

[b22] Ventura S. P. M., Gardas R. L., Goncalves F. & Coutinho J. A. P. Ecotoxicological risk profile of ionic liquids: Octanol-water distribution coefficients and toxicological data. J. Chem. Technol. Biotechnol. 86, 957–963 (2011).

[b23] Kalcikova G., Zagore-Koncan J., Znidarsic-Plazl P. & Gotvajn A. Z. Assessment of environmental impact of pyridinium-based ionic liquid. Fresenius Environ. Bull. 21, 2320–2325 (2012).

[b24] Stolte S. . Ionic liquids as lubricants or lubrication additives: An ecotoxicity and biodegradability assessment. Chemosphere 89, 1135–1141 (2012). 22749125 10.1016/j.chemosphere.2012.05.102

[b25] Ventura S. P. M. . Toxicity assessment of various ionic liquid families towards vibrio fischeri marine bacteria. Ecotox. Environ. Safe. 76, 162–168 (2012). 10.1016/j.ecoenv.2011.10.006 22019310

[b26] Viboud S. . Correlating the structure and composition of ionic liquids with their toxicity on vibrio fischeri: A systematic study. J. Hazard. Mater. 215, 40–48 (2012). 22417395 10.1016/j.jhazmat.2012.02.019

[b27] Ventura S. P. . Designing ionic liquids: The chemical structure role in the toxicity. Ecotoxicology 22, 1–12 (2013). 23010869 10.1007/s10646-012-0997-x

[b28] Stolte S., Schulz T., Cho C. W., Arning J. & Strassner T. Synthesis, toxicity, and biodegradation of tunable aryl alkyl ionic liquids (taails). ACS Sustain. Chem. Eng. 1, 410–418 (2013).

[b29] Ventura S. P. M. . Imidazolium and pyridinium ionic liquids from mandelic acid derivatives: Synthesis and bacteria and algae toxicity evaluation. ACS Sustain. Chem. Eng. 1, 393–402 (2013).

[b30] Carvalho P. J. . Understanding the impact of the central atom on the ionic liquid behavior: Phosphonium vs ammonium cations. Chem. Phys. Lipids 140, 064505 (2014). 10.1063/1.4864182 24527930

[b31] Kurnia K. A. . The effect of the cation alkyl chain branching on mutual solubilities with water and toxicities. Phys. Chem. Chem. Phys. 16, 19952–19963 (2014). 25119425 10.1039/c4cp02309a PMC4265389

[b32] Silva F. A. E. . Sustainable design for environment-friendly mono and dicationic cholinium-based ionic liquids. Ecotox. Environ. Safe. 108, 302–310 (2014). 10.1016/j.ecoenv.2014.07.003 25108510

[b33] Ventura S. P. M. . Ecotoxicity analysis of cholinium-based ionic liquids to vibrio fischeri marine bacteria. Ecotox. Environ. Safe. 102, 48–54 (2014). 10.1016/j.ecoenv.2014.01.003 24580821

[b34] Costa S. P. F., Pinto P., Lapa R. A. S. & Saraiva M. Toxicity assessment of ionic liquids with vibrio fischeri: An alternative fully automated methodology. J. Hazard. Mater. 284, 136–142 (2015). 25463227 10.1016/j.jhazmat.2014.10.049

[b35] Hernández-Fernández F. J. . Discovering less toxic ionic liquids by using the microtox^®^ toxicity test. Ecotox. Environ. Safe. 116, 29–33 (2015). 10.1016/j.ecoenv.2015.02.034 25748519

[b36] Wang C., Wei Z., Wang L., Sun P. & Wang Z. Assessment of bromide-based ionic liquid toxicity toward aquatic organisms and qsar analysis. Ecotox. Environ. Safe. 115, 112–118 (2015). 10.1016/j.ecoenv.2015.02.012 25682588

[b37] Montalbán M. G., Hidalgo J. M., Collado-González M., Díaz Baños F. G. & Víllora G. Assessing chemical toxicity of ionic liquids on vibrio fischeri: Correlation with structure and composition. Chemosphere 155, 405–414 (2016). 27139120 10.1016/j.chemosphere.2016.04.042

[b38] Oliveira M. V. S. . (eco)toxicity and biodegradability of protic ionic liquids. Chemosphere 147, 460–466 (2016). 26796340 10.1016/j.chemosphere.2015.11.016

[b39] Perales E. . Comparative ecotoxicology study of two neoteric solvents: Imidazolium ionic liquid vs. Glycerol derivative. Ecotox. Environ. Safe. 132, 429–434 (2016). 10.1016/j.ecoenv.2016.05.021 27265564

[b40] Lechuga M., Fernández-Serrano M., Jurado E., Núñez-Olea J. & Ríos F. Acute toxicity of anionic and non-ionic surfactants to aquatic organisms. Ecotox. Environ. Safe. 125, 1–8 (2016). 10.1016/j.ecoenv.2015.11.027 26650419

[b41] Egorova K. S. & Ananikov V. P. Toxicity of ionic liquids: Eco(cyto)activity as complicated, but unavoidable parameter for task-specific optimization. ChemSusChem 7, 336–360 (2014). 24399804 10.1002/cssc.201300459

[b42] Gal N. . Membrane interactions of ionic liquids: Possible determinants for biological activity and toxicity. Biochim. Biophys. Acta - Biomembranes 1818, 2967–2974 (2012). 10.1016/j.bbamem.2012.07.025 22877704

[b43] Biltonen R. L. & Lichtenberg D. The use of differential scanning calorimetry as a tool to characterize liposome preparations. Chem. Phys. Lipids 64, 129–142 (1993).

[b44] Chiu M. & Prenner E. Differential scanning calorimetry: An invaluable tool for a detailed thermodynamic characterization of macromolecules and their interactions. J. Pharm. Bioallied Sci. 3, 39–59 (2011). 21430954 10.4103/0975-7406.76463 PMC3053520

[b45] Majhi P. R. & Blume A. Temperature-induced micelle-vesicle transitions in dmpc−sds and dmpc−dtab mixtures studied by calorimetry and dynamic light scattering. J. Phys. Chem. B. 106, 10753–10763 (2002).

[b46] López O. . Direct formation of mixed micelles in the solubilization of phospholipid liposomes by triton x-100. FEBS Letters 426, 314–318 (1998). 9600258 10.1016/s0014-5793(98)00363-9

[b47] Inoue T., Yanagihara S.-i., Misono Y. & Suzuki M. Effect of fatty acids on phase behavior of hydrated dipalmitoylphosphatidylcholine bilayer: Saturated versus unsaturated fatty acids. Chem. Phys. Lipids 109, 117–133 (2001). 11269932 10.1016/s0009-3084(00)00170-5

[b48] Mukerjee P. & Mysels K. J. Critical micelle concentrations of aqueous surfactant systems. (U.S. Department of Commerce, National Bureau of Standards, 1971).

[b49] Campbell A. N. & Lakshminarayanan G. R. Conductances and surface tensions of aqueous solutions of sodium decanoate, sodium laurate, and sodium myristate, at 25° and 35°. Can. J. Chem. 43, 1729–1737 (1965).

