# Supplementary material for: Impact of Surface-Active Guanidinium-, Tetramethylguanidinium-, and Cholinium-Based Ionic Liquids on Vibrio Fischeri Cells and Dipalmitoylphosphatidylcholine Liposomes
Source: Sci Rep. 2017 Apr 21;7:46673. doi: 10.1038/srep46673 (PMC5399364; doi:10.1038/srep46673)

IMPACT OF SURFACE-ACTIVE GUANIDINIUM-, TETRAMETHYLGUANIDINIUM-, AND CHOLINIUM-BASED IONIC LIQUIDS ON *VIBRIO FISCHERI* CELLS AND DIPALMITOYLPHOSPHATIDYLCHOLINE LIPOSOMES

Supplementary information

Antti H. Rantamäki[1], Suvi-Katriina Ruokonen[1], Evangelos Sklavounos[1], Lasse Kyllönen[2], Alistair W. T. King,[1]* Susanne K. Wiedmer[1]*

1) Department of Chemistry, POB 55, 00014 University of Helsinki, Finland

2) Kemira Oyj, Luoteisrinne 2, P.O.Box 44, FI-02271 Espoo, Finland

SUPPLEMENTARY MATERIAL

^{1}H and ^{13}C NMR Spectra (600 MHz) of synthesized ionic liquids

Neodecanoic Acid - Versatic™ Acid (DMSO-d_6)

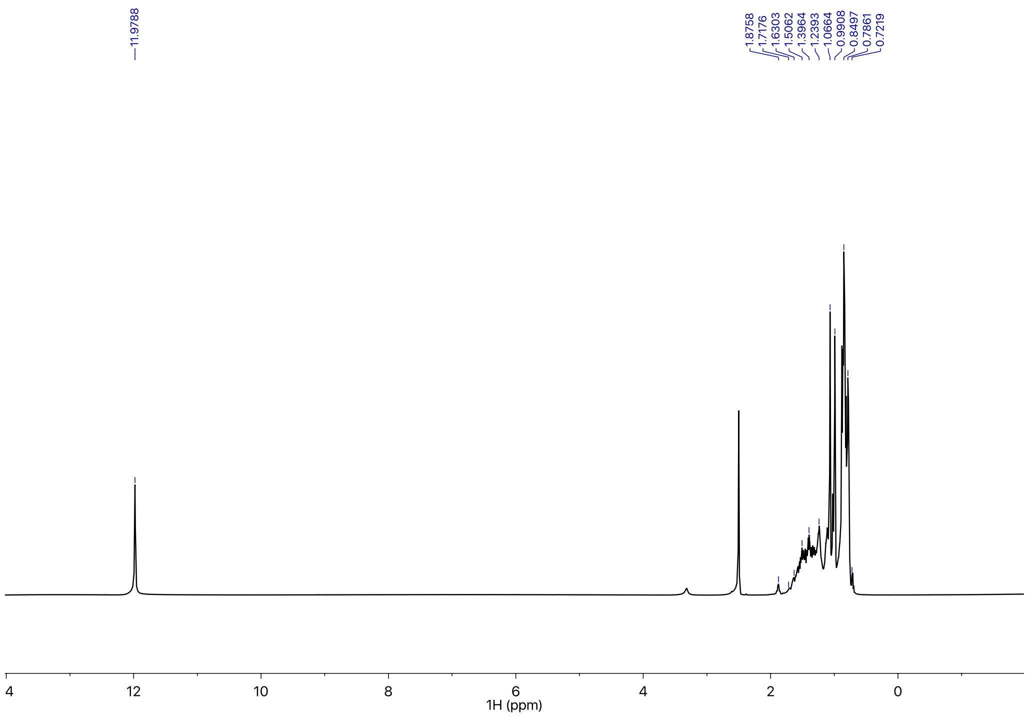

Isostearic Acid – Pristorine™ 3501 (DMSO-d_6, with 1,4-dioxane as internal standard)

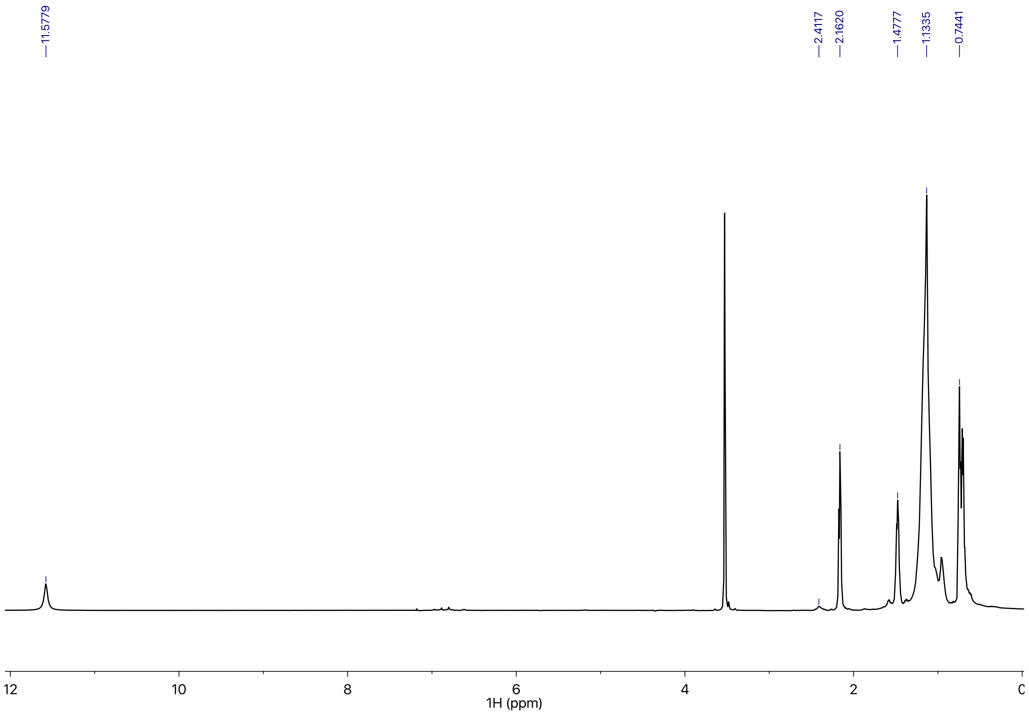

Choline Isostearate (D_2O), gel-like liquid

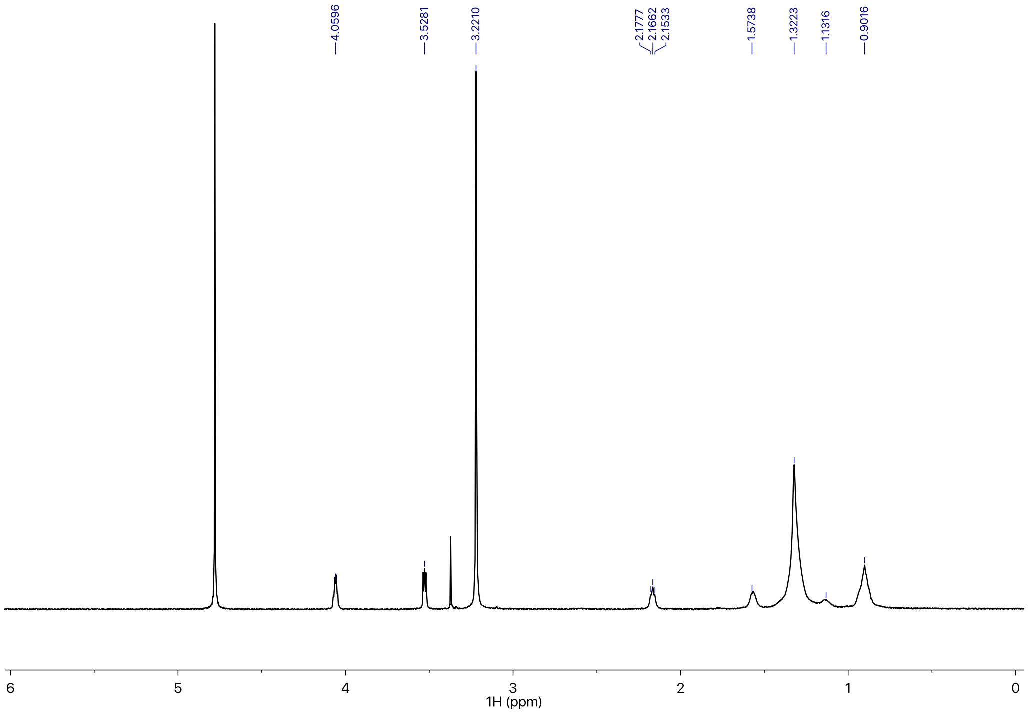

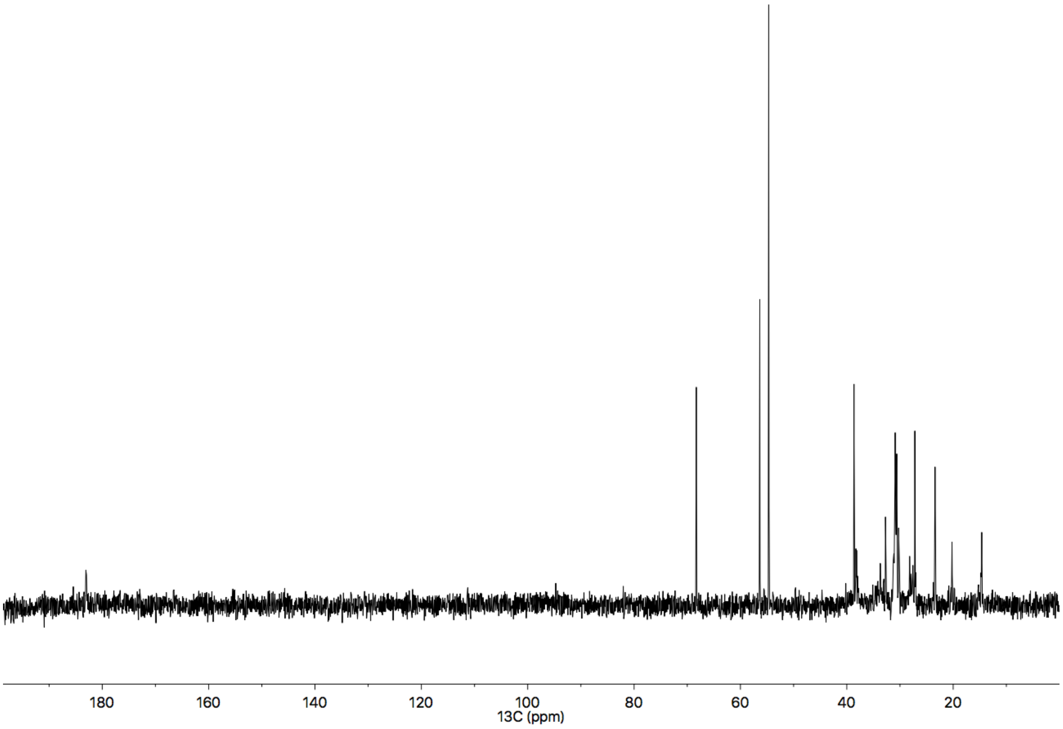

Choline Decanoate (D_2O), melting point = 3 °C.

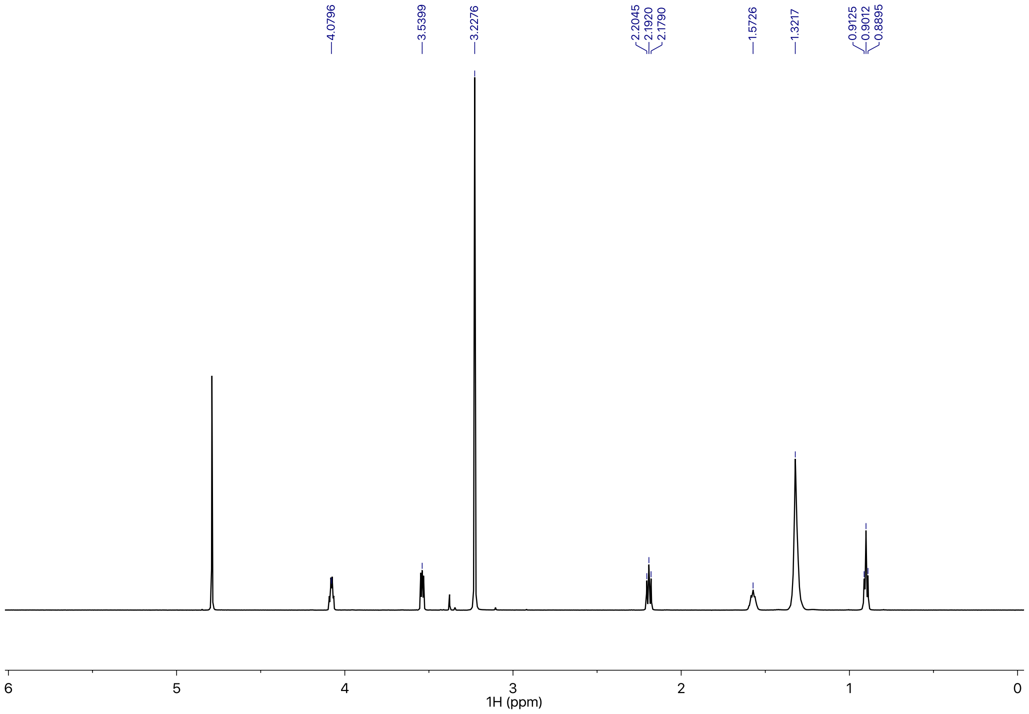

Choline Neodecanoate (DMSO-d_6), liquid

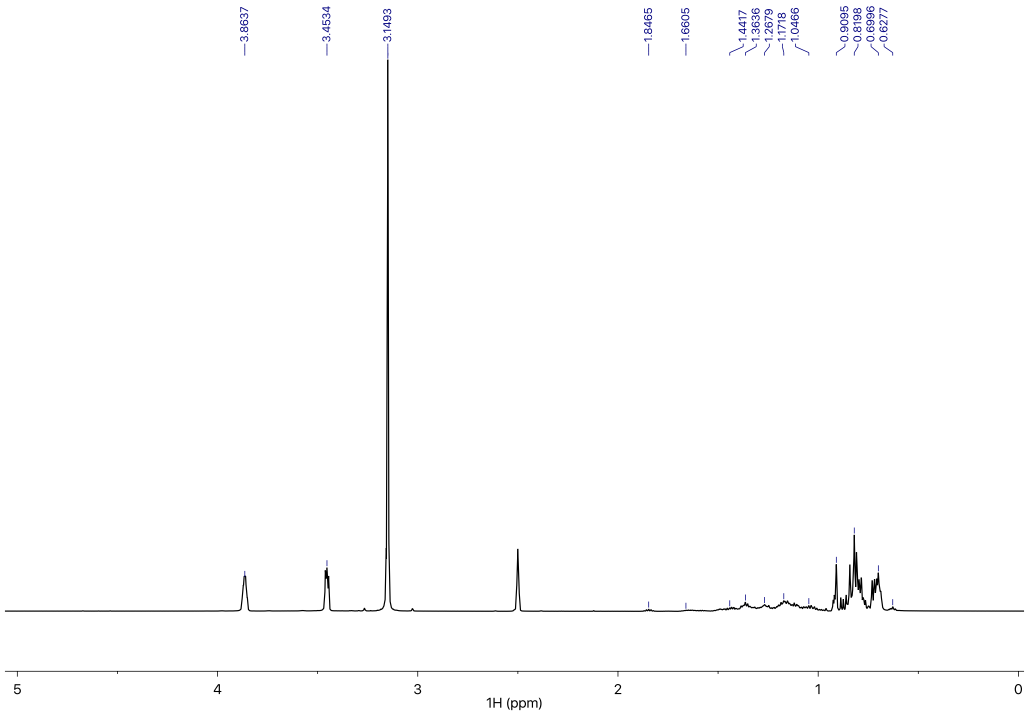

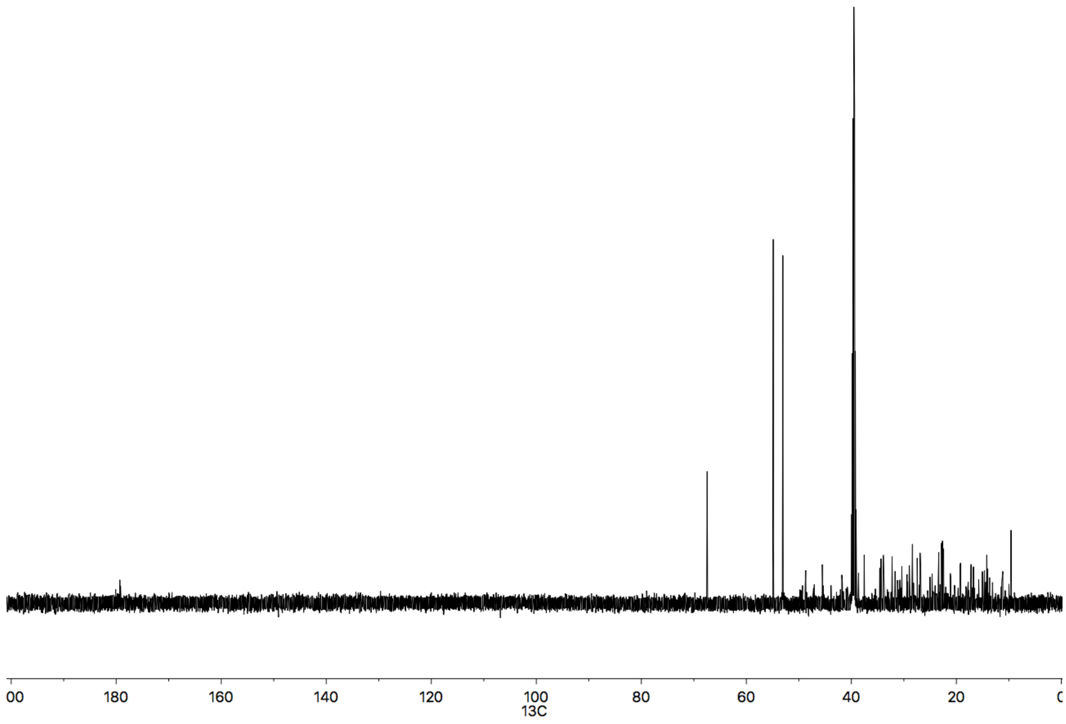

Guanidinium Isostearate (DMSO-d_6), melting point = 76 $^{\circ}$C.

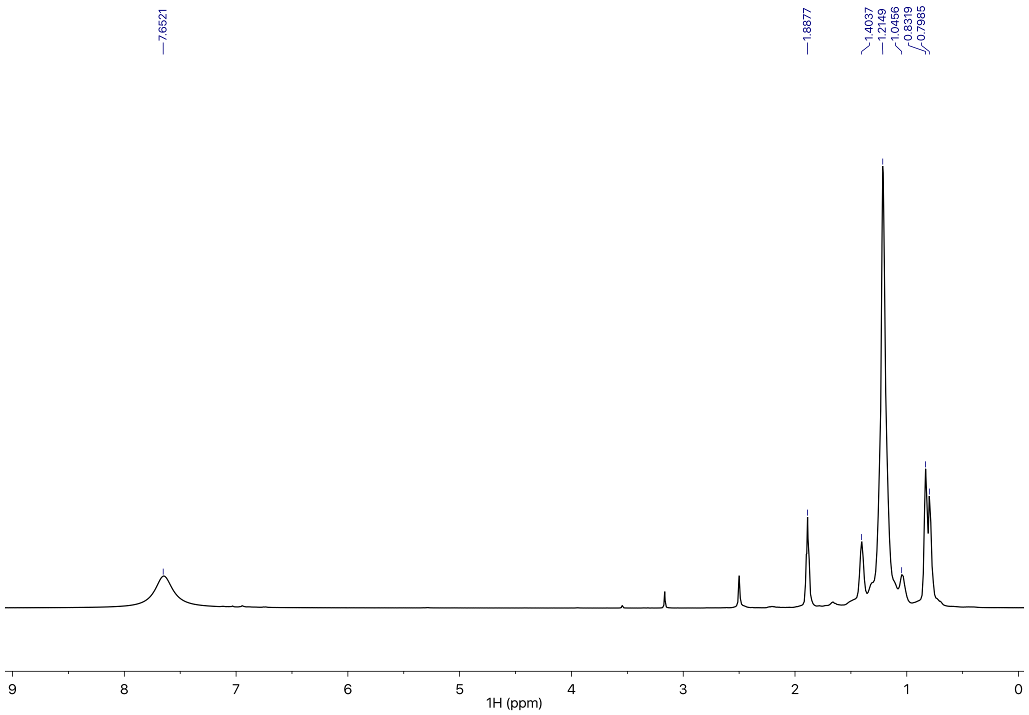

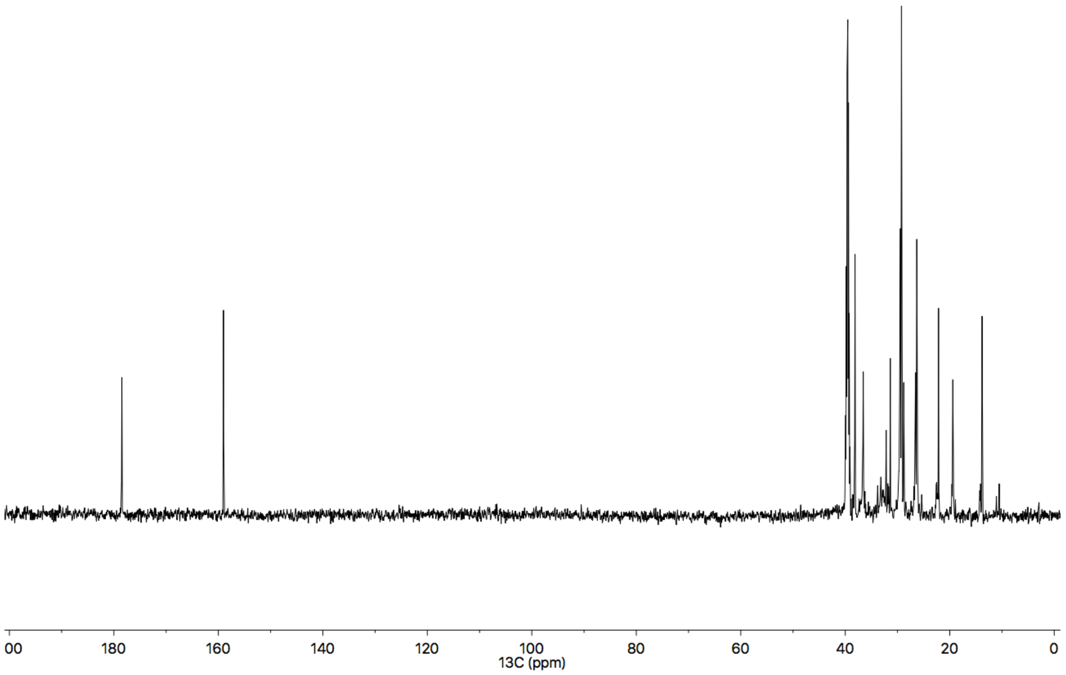

Guanidinium Neodecanoate (DMSO-d_6), Glass transition = 44 $^{\circ}$C.

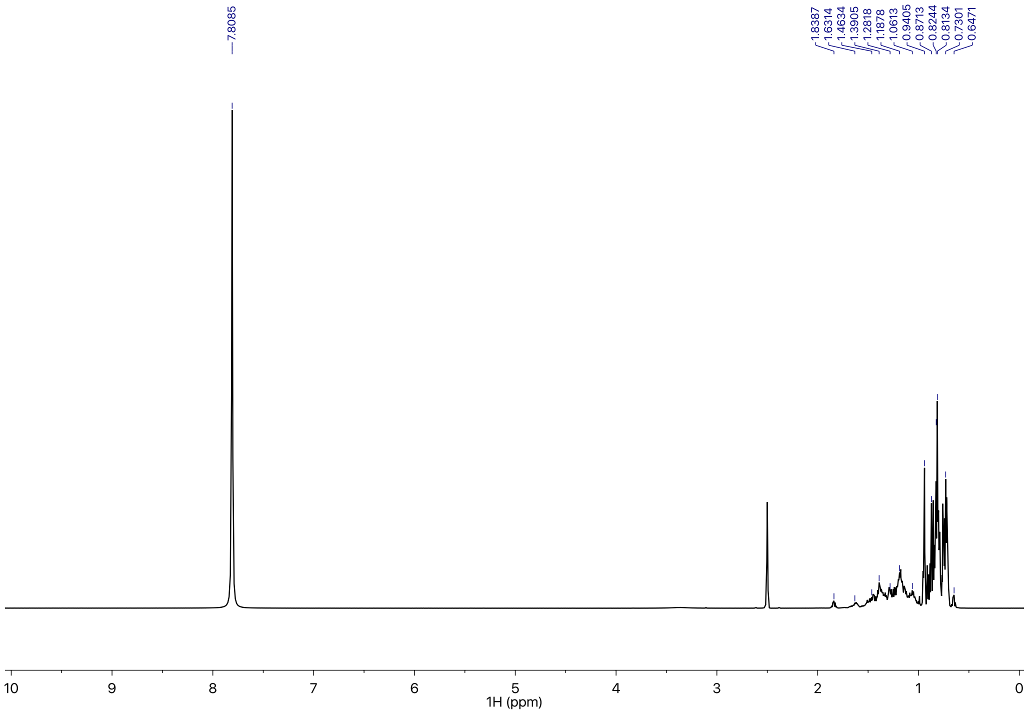

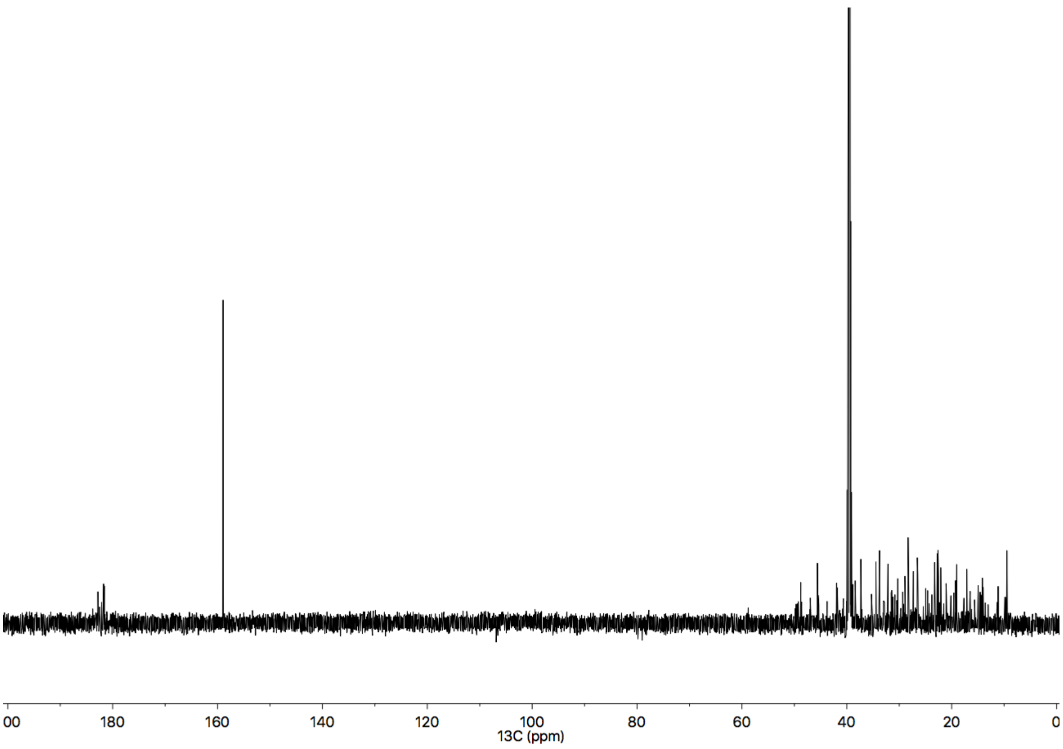

1,1,3,3-Tetramethylguanidinium Isostearate (D_2O), melting point = 24 °C.

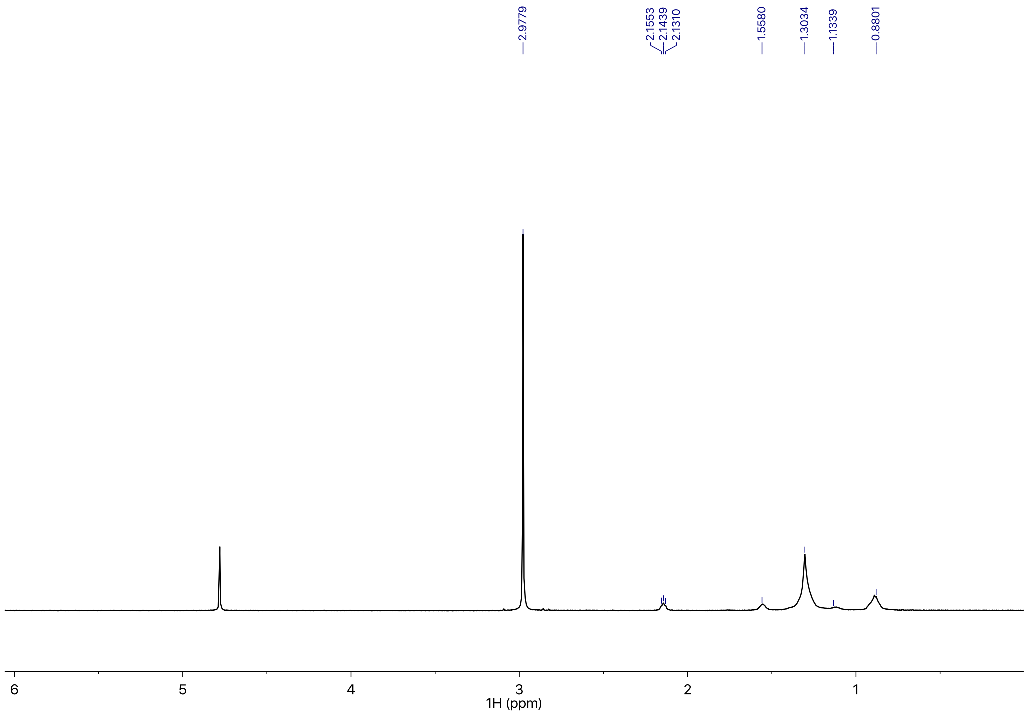

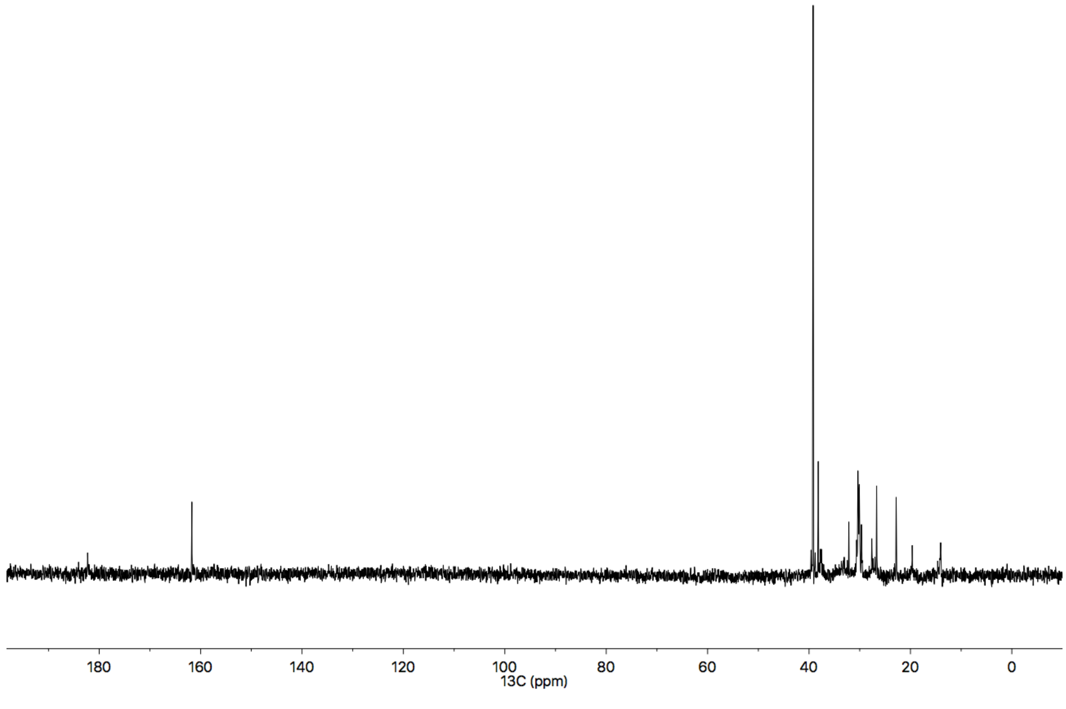

1,1,3,3-Tetramethylguanidinium Neodecanoate (DMSO-d_6), liquid.

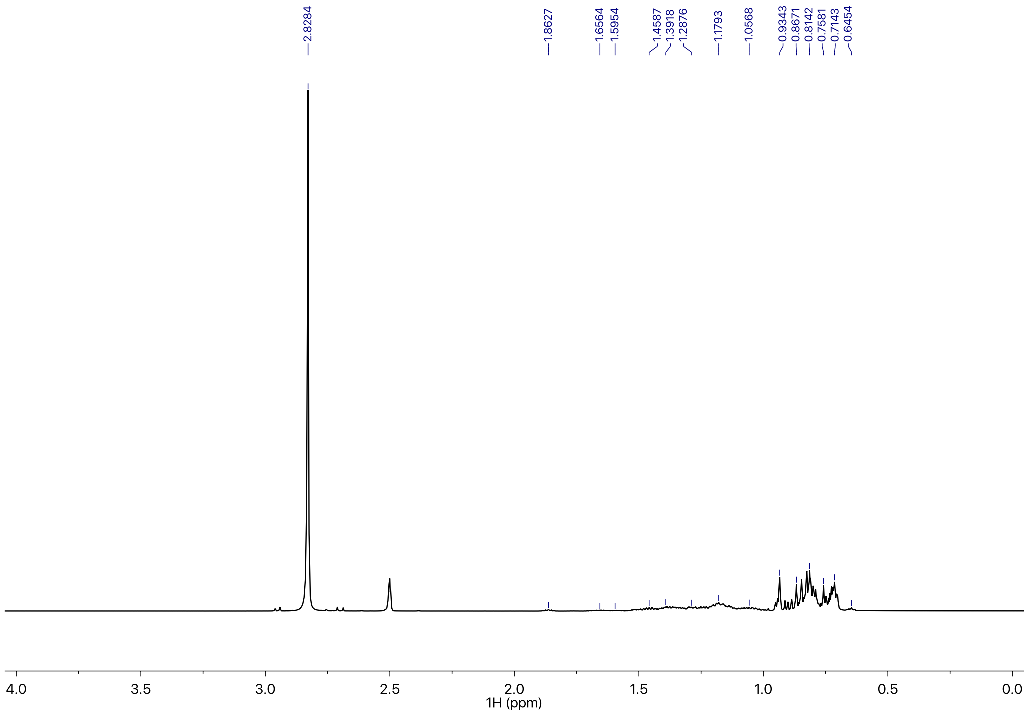

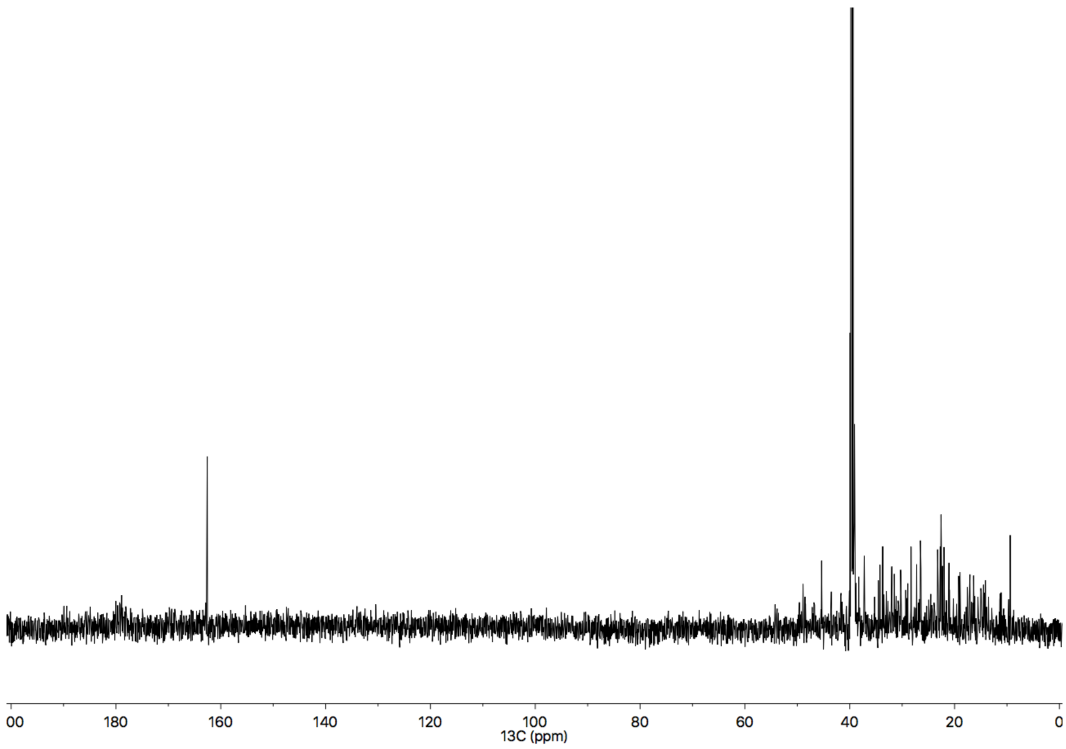

Sodium Isostearate (D_2O)

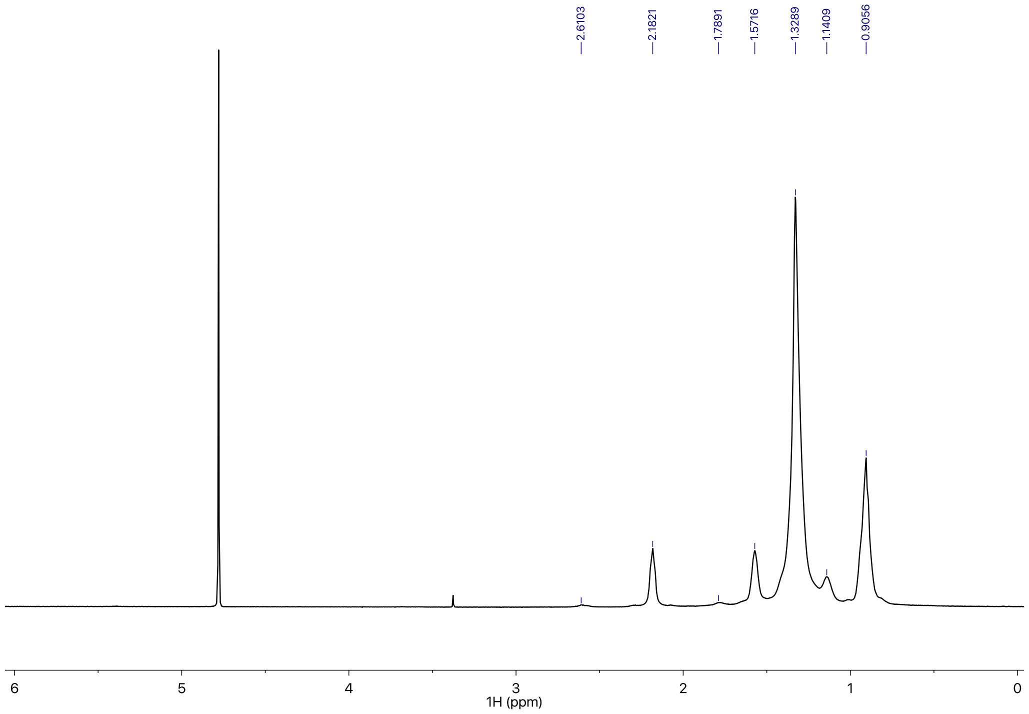

Sodium Neodecanoate (DMSO-d_6)

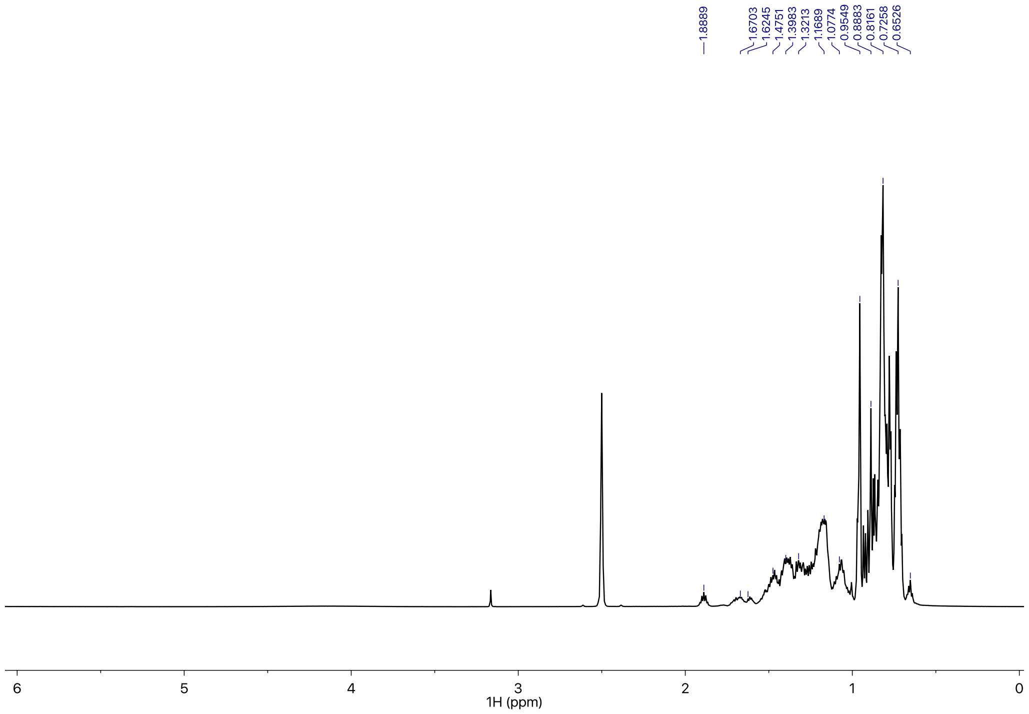

Supplement: Supplementary Information [file srep46673-s1.pdf]
